# Editorial: Music performance anxiety, volume II

**DOI:** 10.3389/fpsyg.2026.2007842

**Published:** 2026-10-12

**Authors:** Oscar Casanova, Michiko Yoshie, Katarina Habe, Patrick Gomez

**Affiliations:** 1 Department of Musical, Plastic and Corporal Expression, Faculty of Education, University of Zaragoza, Zaragoza, Spain; 2 Human Informatics and Interaction Research Institute, National Institute of Advanced Industrial Science and Technology (AIST), Tsukuba, Japan; 3 Department of Human and Engineered Environmental Studies, Graduate School of Frontier Sciences, The University of Tokyo, Kashiwa, Japan; 4 Institute of Human Sciences, University of Tsukuba, Tsukuba, Japan; 5 Academy of Music, University of Ljubljana, Ljubljana, Slovenia; 6 Department of Occupational and Environmental Health, Unisanté, University Center for Primary Care and Public Health and University of Lausanne, Lausanne, Switzerland

**Keywords:** coping strategies, digital performance anxiety, music performance anxiety, musician wellbeing, performance simulation, psychometric validation, self-efficacy, stage fright

## 1 Introduction

Music performance anxiety (MPA) is a complex psychological and physiological phenomenon that affects musicians of all ages, genders, and experience levels, from adolescent learners to internationally renowned soloists. Traditionally, scientific literature has largely approached this phenomenon from clinical or pathological viewpoints, frequently equating it with social phobia or generalized anxiety disorder. However, the contemporary era has witnessed a profound conceptual and methodological paradigm shift. This is clearly evident in the recent Research Topic of scientific articles published on this specialized topic.

The purpose of this editorial is to synthesize these findings systematically, providing a clear conceptual roadmap for researchers, educators, and performers. This document is organized around five thematic and methodological axes, reviewing how recent studies structure the modern understanding of MPA. Together, these axes aim to depathologize performance-related stress and propose a comprehensive psychological framework that enables musicians to flourish artistically, not merely endure the stage.

## 2 Theoretical advances, quantitative methodology, and instrument validation

For decades, the relationship between physiological arousal and musical execution was assumed to follow the classic inverted-U curve (derived from the Yerkes–Dodson law), which posits that both extremely low and excessively high levels of arousal impair performance, while moderate activation remains optimal. Challenging this unidimensional perspective, Twitchell et al. reconceptualize MPA in light of advances in affective science over the past 20 years. The authors propose applying the Biopsychosocial Model of Challenge and Threat, arguing that the impact of MPA is not determined by the absolute quantity of stress, but rather by the performer's cognitive appraisal. When perceived situational demands exceed available coping resources, a threat response is triggered, characterized by increased total peripheral resistance, cortisol elevation, and avoidance motivation. Conversely, when the musician appraises their resources as sufficient to meet the demands, a challenge response is elicited, leading to cardiovascular efficiency, optimal resource mobilization, and approach motivation.

To measure these fluctuating states during live performance, contemporary researchers have developed and validated several context-sensitive, cross-cultural psychometric scales. Spahn and Nusseck introduce and validate the German Performance-related Questionnaire for Musicians (PQM) in a substantial sample of 605 musicians. Unlike traditional inventories that measure MPA as a stable, situation-independent trait, the PQM retrospectively evaluates three distinct temporal phases (before, during, and immediately after a concert) across three primary dimensions: symptoms of MPA, functional coping, and situational self-efficacy. The authors demonstrate that the PQM is highly sensitive to state-level fluctuations, serving as a practical diagnostic tool. The factor structure and validity of this instrument were further confirmed in a Slavic context by Kaleńska-Rodzaj et al., using a sample of 258 Polish soloists, choral singers, and orchestral musicians. Through confirmatory factor analysis, the researchers demonstrated the robustness of the three situational dimensions and their positive correlation with flow experience and generalized self-efficacy, establishing strong cross-cultural validity.

In Southern Europe, Barros et al. developed and validated the Portuguese Music Performance Anxiety Scale (PoMPAS) with a sample of 414 higher education music students. Anchored in traditional tri-dimensional models (somatic, cognitive, and behavioral), the PoMPAS demonstrated excellent internal consistency and revealed a robust three-factor structure: a behavioral/emotional factor, a contextual/physiological factor, and a cognitive factor. This scale represents the first psychometric instrument developed specifically for higher music education in Portugal, integrating specific performance-related context variables such as orchestral auditions and jury evaluations.

The imperative to revalidate and refine existing instruments for specific sub-populations is further exemplified by Pan et al., who re-evaluated the Kenny Music Performance Anxiety Inventory–Revised in a sample of 736 Chinese university vocal students majoring in lyric singing. Recognizing that prior validation studies failed to differentiate between vocalists and instrumentalists, they conducted exploratory and confirmatory factor analyses. Their results yielded a robust four-factor model: Psychological Vulnerability, Proximal Somatic and Cognitive Anxiety, Parental Support, and Memory Self-Efficacy. The authors conclude that vocalists, because they use their own bodies as instruments, experience unique physical and emotional exposure that demands a highly nuanced interpretation of the scale, especially when examined through the lens of traditional Confucian values.

## 3 Intrapersonal, cognitive, and personality factors

The experience of MPA is deeply interconnected with intrapersonal and cognitive variables that mediate or moderate wellbeing and performance quality. Among these constructs, musical self-efficacy plays a primary regulatory role. Ou and Qin offer a crucial contribution by differentiating between two dimensions of self-efficacy in a sample of 254 undergraduate violin students from 11 Chinese conservatories: Music Learning Self-Efficacy (MLSE) and Music Performance Self-Efficacy (MPSE). Their multiple linear regression analysis revealed a compelling, counterintuitive finding: while MPSE is negatively associated with performance-related anxiety (serving as a protective factor that promotes stable execution), MLSE displayed a significant positive association with MPA. The authors interpret this phenomenon by hypothesizing that students with high learning self-efficacy tend to impose excessively high self-expectations and rigid perfectionistic standards, thereby increasing their vulnerability to cognitive interference and sensitivity to perceived failure during public execution.

Furthermore, Borges et al. (a) systematically map the relationship between flow and MPA through a scoping review of 18 empirical studies. Following PRISMA-ScR guidelines, they find that while flow and anxiety are predominantly negatively correlated, they are not strictly antagonistic. Under specific cognitive appraisals, moderate physiological arousal can coexist with or even facilitate flow, provided the musician appraises the situation as a challenge rather than a threat, and maintains an optimal balance between task demands and personal skills (challenge-skill balance). The authors identify self-efficacy, emotional self-regulation, and self-esteem as key mediating variables that facilitate flow propensity and mitigate the debilitating effects of stage fright.

This configuration of positive resources is explored empirically by Jiang and Tong, who investigate the impact of Psychological Capital (PsyCap) in a sample of 329 Chinese conservatory students. PsyCap is a multidimensional construct comprising four positive psychological resources: self-efficacy, optimism, hope, and resilience. Using structural equation modeling, they validate a chain mediation model showing that PsyCap reduces MPA indirectly through two key mediators: self-esteem and flow experience. Specifically, students with higher PsyCap exhibit greater self-esteem, which enhances their propensity to enter flow states during performance, ultimately leading to a significant reduction in debilitating MPA symptoms.

Yang et al. investigate a moderated mediation model of MPA among 609 Chinese music majors, examining whether perceived performance risk mediates the relationship between professional competence and MPA, and whether psychological resilience moderates this pathway. Their analysis reveals that greater technical competence is associated with lower perceived risk and lower MPA. Crucially, psychological resilience acts as a powerful moderator: the protective effect of professional competence on reducing perceived risk is significantly stronger for students with low to moderate resilience. For highly resilient students, this protective effect stabilizes, suggesting that resilience serves as an essential psychological buffer that protects performers under conditions of lower technical preparation.

This resilience framework is further enriched by Du et al. in a study of 801 Chinese community choir members. They model the relationship between resilience and MPA through two mediators: performance control sense and pre-performance rumination. Structural equation modeling demonstrated that resilience is negatively associated with MPA, with performance control sense acting as the dominant mediator. Multi-group analyses revealed notable gender differences: the protective function of control beliefs is more pronounced among women, whereas pre-performance rumination exerts a significantly more debilitating effect on men.

## 4 Social, familial, and digital age influences

The development and manifestation of MPA do not occur in an intrapersonal vacuum, but are deeply shaped by social, familial, and digital environments. Maoqing evaluates the role of perceived social support from parents, educators, and peers in a sample of 526 Chinese vocal students. Using gender-based structural equation modeling, the study confirms that perceived support reduces MPA indirectly by bolstering personal resources, such as self-efficacy, self-worth, and intrinsic motivation. Significant gender differences emerged: female students exhibited substantially stronger indirect effects, suggesting that women are highly receptive to relational support and link their self-worth more closely to socio-emotional backing.

Similarly, Huawei and Jenatabadi explore social support in 483 Chinese undergraduate and postgraduate music students. Grounded in Stimulus-Organism-Response theory, they validate a model in which emotional intelligence and self-efficacy mediate the relationship between social support and MPA. The analysis highlights educational level differences: postgraduate students show stronger mediating effects, suggesting that academic maturity and performance experience enhance the capacity to internalize and utilize supportive social and emotional resources.

In collective performance settings, Wu et al. examine gender disparities in MPA in relation to evaluative scrutiny among 774 Chinese choir singers. The authors distinguish between self-scrutiny (internal focus on rigid standards and post-performance rumination) and other-scrutiny (worry about judgment from the audience, conductor, or peers). Their structural model reveals that female singers report significantly higher MPA, and that self-scrutiny accounts for the largest proportion of the indirect pathway. They conclude that internalized evaluative cognitive styles represent the dominant mechanism explaining gender-based differences in performance-related anxiety.

The rise of digital performance platforms and social media has introduced novel sources of evaluative stress. Ma et al. employed structural equation modeling to investigate online evaluation anxiety in a sample of 320 conservatory students engaged in digital performance. Their model demonstrates that digital performance exposure positively predicts online evaluation anxiety, which subsequently leads to higher levels of MPA than those produced in traditional contexts (on stage; not digital). This mediating pathway is moderated by two contrasting individual traits: perfectionism (which amplifies online evaluation anxiety) and self-compassion (which serves as a psychological buffer, weakening the link between evaluative stress and MPA).

Contextual demands also manifest within the professional workspace. Babnik et al. investigated the relationship between job characteristics (demands and resources) and MPA in 94 collaborative pianists employed in Slovenian music schools. Multiple linear regression revealed that perceived job characteristics explained 50% of the variance in MPA. Perceived emotional demands (particularly, the need to suppress personal opinions and emotions to maintain interpersonal harmony) and role conflict emerged as the strongest predictors of performance anxiety. The authors conclude that collaborative pianists' MPA is not merely an individual technical issue, but a systemic symptom of organizational stress and ambiguous role boundaries relative to primary instrumental teachers.

To explore how individual and environmental factors interact under different situational conditions, Bao and Yu conducted a two-study investigation. Study 1 (correlational, *N* = 1,364 Chinese conservatory students) showed that perfectionism and collectivist orientation significantly moderate the relationship between performance anxiety and wellbeing (measured via the PERMA model). Study 2 (experimental, *N* = 218) randomly assigned participants to individual success, collaborative success, or neutral conditions. The experiment revealed that individual success conditions—which impose rigid perfectionist standards—elicited the highest levels of anxiety. However, these individual conditions also generated higher wellbeing scores related to personal accomplishment. This illustrates a compelling paradox: high-pressure individual performance contexts may elevate anxiety but concurrently provide intense psychological gratification when artistic excellence is achieved. This is complemented by Takagi et al., who examined correlates of MPA in 390 Japanese professional and amateur musicians. Their multivariable model shows that attending specialized music schools (which impose early evaluative pressure) positively predicts MPA, whereas cumulative lifetime public performance count is negatively associated with anxiety, supporting behavioral habituation and extinction mechanisms.

## 5 Clinical and pedagogical interventions, and cutting-edge technologies

The preventive and therapeutic management of MPA has undergone significant diversification, combining traditional behavioral therapies with cutting-edge technological innovations. Bakhtiari et al. conducted a systematic review of coping and intervention strategies implemented between 2016 and 2023, analyzing thirteen high-quality studies (using the Newcastle-Ottawa Scale). Their synthesis demonstrates that Cognitive Behavioral Therapy (CBT) and Acceptance and Commitment Therapy (ACT) are highly effective in reducing cognitive symptoms and enhancing psychological flexibility. Concurrently, somatic approaches, such as yoga and meditation, significantly attenuate sympathetic nervous system arousal, validating the need for multi-faceted interventions that integrate mind and body techniques tailored to individual performers.

Regarding brief, accessible interventions, Paese and Schiavio evaluated a 4-week mindfulness course with twelve music students and professionals at the University of York. Practice diaries and qualitative data revealed that body-centered meditation, Vipassana (mindfulness of thoughts), and the affect-centered RAIN technique (Recognize, Allow, Investigate, and Nurture) progressively fostered self-compassion and non-judgmental awareness of muscle tension. Participants reported that these practices deactivated mental rumination, restored their artistic connection to the instrument, and mitigated somatic pre-performance panic. Similarly, Bojner Horwitz and Valtasaari developed a practical, self-administered manual consisting of eighteen steps illustrated with stick figures. Tested in a qualitative case study, this manual—grounded in embodied knowledge and mindfulness—enabled musicians to observe and accept somatic symptoms without catastrophic appraisal, fostering a compassionate response to performance stress.

In vocal pedagogy, Cong assesses the short- and long-term efficacy of a four-stage integrated pedagogical model termed the “Psychological Scaffolding Model” (ASSR: Awareness-Skill-Simulation-Reflection). In a mixed-methods study with a pre-test, post-test, and 3-month follow-up involving 60 undergraduate vocal majors, the experimental group (ASSR) showed a significant reduction in state anxiety, improved vocal performance quality (blind expert ratings), increased psychological resilience, and enhanced heart rate variability. Qualitative analysis revealed deep transformational shifts across four dimensions: cognitive restructuring, enhanced bodily control (utilizing diaphragmatic breathing as an emotional anchor), reframing the stage experience, and developing a trusting, collaborative teacher-student alliance.

Emerging technologies and simulated environments offer promising avenues for performance preparation under controlled conditions. Borges et al. (b) developed and validated an emotional self-regulation protocol utilizing Neurofeedback combined with Functional Near-Infrared Spectroscopy (NFDK-fNIRS) in three adolescent musicians. The protocol alternated standard auditory-guided blocks of anxiety induction and regulation (controlled via PsychoPy and TurboSatori). The fNIRS data showed increased, stable activation in the left dorsolateral prefrontal cortex and dorsomedial prefrontal cortex across sessions, suggesting the acquisition of neurophysiological top-down cognitive control over limbic responses. Additionally, Waddell et al. detail the launch of the second-generation Performance Simulator at the Royal College of Music (RCM) in London. This facility recreates immersive Mixed Reality environments of three RCM concert halls and features a 64-speaker acoustic enhancement constellation. This system allows musicians to train under pressure in a gradual, controlled manner, facilitating mock auditions (try-outs) and self-regulated learning before facing actual juries or public audiences.

This structured exposure is theoretically supported by de Bie et al. in a narrative review of Pressure Training in music. Analyzing empirical evidence from elite sports and police training, the authors propose that systematic exposure to controlled stress in daily practice serves as a “behavioral vaccination” that prevents choking under pressure in musicians. By manipulating training demands (such as auditory distractions) and performance consequences (such as peer evaluations or incentives), musicians develop attentional redirection strategies, automating technical execution under high emotional demands and complementing traditional anxiety mitigation.

## 6 Career perspectives, teaching contexts, and qualitative approaches

Beyond immediate stage execution, MPA directly influences long-term professional development and career aspirations. Wang and Yang evaluated the impact of MPA on the career expectations of 340 Chinese high school music majors. Regression analyses demonstrated that MPA correlates negatively with self-efficacy and future career expectations (measured via the Career Futures Inventory). However, self-efficacy emerged as a critical partial moderator: students with a robust sense of self-efficacy maintained high career aspirations and optimism despite experiencing elevated MPA, highlighting the necessity of curricula and pedagogies that build personal efficacy beliefs from an early stage.

This dual professional identity is also analyzed in preservice music teachers, who navigate roles as both performers and educators. Cui et al. explore the relationships between MPA, Teaching Anxiety (TA), and self-efficacy in a sample of 237 Chinese university students. Stepwise regression demonstrated that self-efficacy negatively predicts both MPA and TA. Furthermore, TA emerged as the strongest predictor of MPA, suggesting a deep interconnection between the fear of instrumental evaluation and the fear of pedagogical evaluation in the classroom. The authors recommend that teacher-training institutions design integrated interventions that address both performance and teaching anxieties concurrently.

Qualitative research utilizing life histories provides access to the subjective depth and existential meaning of performance anxiety. Casanova et al. employed Interpretative Phenomenological Analysis to explore the life histories of four Spanish classical musicians. Their testimonies revealed a shared trajectory: early musical training was associated with play, enjoyment, and positive social support from family and peers. However, as they transitioned to advanced levels with intensifying technical demands, the pressure of jury evaluations and the fear of memory failure or errors transformed music-making into a source of distress and somatic tension. Notably, two of the four participants abandoned their higher music studies before entering university due to the severity of their anxiety, highlighting a pedagogical gap in conservatoires. To address this, the musicians recommend adopting empathetic teaching styles, flexible goal-setting, and the promotion of artistic creativity.

Lim and Dhamabutra, presenting a critical perspective grounded in psychological science and performance practice, argue that traditional symptom-reduction therapies fail to resolve the core causes of anxiety. They assert that when musicians prepare effectively using deep learning strategies, anxiety itself does not compromise stage accuracy. They propose two evidence-based strategies: retrieval practice and non-serial practice. Non-serial practice involves segmenting musical scores and practicing starting from random, intermediate bars to break linear serial associative chains in the brain. This strengthens content-addressable memory access, protecting the performer against memory slips under high evaluative stress.

This holistic, existential perspective is further developed by Šimunovič through the concept of the “Orpheus complex.” The author proposes that stage fright should be interpreted not as an isolated clinical disorder, but as a symptom of asymmetry in the musical self-concept. The artistic manifestation is structured around a tripartite schema: the relationship with oneself, the relationship with others, and the relationship with the transcendent dimension of art. Traditional conservatory pedagogy often hyper-focuses on individual technical perfection and audience evaluation, neglecting the transcendent vertex of musical creation. Šimunovič advocates for rebalancing the musician's self-concept, integrating ritual and transcendence as a protective shield that depathologizes anxiety and restores the liberating purpose of musical art.

## 7 Conclusions

The compiled research shows that MPA should be viewed as a dynamic, multidimensional biopsychosocial phenomenon that goes beyond individual clinical diagnoses. The reviewed empirical and methodological advances reveal a clear transition from symptom reduction and pharmacological mitigation toward cognitive optimization, pedagogical scaffolding, and technological innovation. This evolution shows us that performance-related stress is not a pathology to be eradicated, but rather an adaptive resource that can be effectively evaluated and managed.

Furthermore, personal resources such as performance-specific self-efficacy, self-esteem, psychological capital, and self-compassion act as powerful protective factors against the demands of the digital era and continuous online evaluation. Implementing integrated preventative programs in conservatories is the most effective way to support the sustainable development and mental health of future musicians. Ultimately, as the myth of Orpheus reminds us, performers must preserve their connection to the transcendent and collective purpose of art. It is the responsibility of institutions, educators, and the scientific community to rebalance educational and professional ecosystems, transforming the stage from a hostile arena of judgment to a shared space of artistic communion and communication.

